# Barriers and facilitators to career progression and leadership among internationally educated nurses and midwives in the UK: a mixed methods systematic review

**DOI:** 10.3389/frhs.2026.1854601

**Published:** 2026-10-21

**Authors:** Rohit Sagoo, Clair Merriman, Tricia Mukherjee, Nyarai Gondo, Sharin Baldwin

**Affiliations:** 1 Corporate Nursing, London North West University Healthcare Trust, London, United Kingdom; 2 School of Nursing and Health Sciences within the Faculty of Health Sciences and Wellbeing, University of Sunderland in London, United Kingdom; 3 Institute for Health and Wellbeing Research, University of Bedfordshire, United Kingdom; 4 Oxford University Hospital NHS Foundation Trust, Oxford, United Kingdom; 5 Oxford School of Nursing and Midwifery, Oxford Brookes University, United Kingdom; 6 Innovation and Research, Institute of Health Visiting, London, United Kingdom; 7 Centre for Health Services Studies, University of Kent, Canterbury, United Kingdom; 8 School of Nursing and Midwifery, Western Sydney University, Penrith, NSW, Australia

**Keywords:** career progression, internationally educated nurses and midwives, leadership, mentorship, organisational cultures, systemic barriers, United Kingdom, workforce development

## Abstract

**Background:**

Internationally educated nurses and midwives (IENMs) comprise almost one-quarter of the UK nursing and midwifery workforce and play a vital role in addressing workforce shortages across the National Health Service (NHS). Despite their significant contribution, evidence suggests that IENMs experience barriers to career progression and leadership development. This review aimed to synthesise evidence on the barriers and facilitators that influence career progression among IENMs working in the United Kingdom.

**Methods:**

A mixed-methods systematic review was conducted using the Joanna Briggs Institute (JBI) methodology. Five electronic databases (CINAHL, MEDLINE, PubMed, Cochrane Library, and Embase) were searched, along with grey literature sources, for studies published between January 2005 and March 2025. Eligible studies included qualitative, quantitative, and mixed methods research involving internationally educated nurses and/or midwives practising in the UK. Methodological quality was assessed using the JBI Critical Appraisal Checklist for Qualitative Research and the Mixed Methods Appraisal Tool (MMAT). Quantitative findings were transformed into qualitative data and integrated with qualitative findings using the JBI convergent integrated approach.

**Results:**

Eight studies met the inclusion criteria, representing at least 1,752 participants. Thirty-two qualitative findings and eight qualitised findings were synthesised into five integrated themes: (1) Career Progression and Structural Barriers; (2) Early-Career Scaffolding and Development Support; (3) Skill Mismatch and De-skilling; (4) Mentoring, Sponsorship and Leadership Access; and (5) Organisational Culture and Inclusion. IENMs experienced delayed promotion, limited recognition of prior expertise, unequal access to professional development, and inconsistent mentorship and sponsorship. Facilitators included structured induction, supportive line management, leadership development programmes, equitable access to continuing professional development, and inclusive organisational cultures. The evidence suggested that career progression was influenced more by organisational and structural factors than by individual capability.

**Conclusion:**

IENMs continue to face significant systemic barriers to career progression and leadership development within the UK healthcare workforce. Addressing these challenges requires transparent promotion pathways, recognition of international experience, equitable access to development opportunities, and targeted mentorship and sponsorship initiatives. Strengthening inclusive organisational cultures and leadership practices may improve retention, workforce sustainability, and leadership diversity within the NHS and wider healthcare sector.

**Systematic Review Registration:**

https://www.crd.york.ac.uk/PROSPERO/view/CRD420251006645.

## Introduction

There are over 800,000 nurses and midwives registered in the United Kingdom (UK) (1), of which Internationally Educated Nurses (IENs) and Midwives (IEMs) make up 23.8% of the total nursing and midwifery workforce (2). IENs and IEMs are crucial in sustaining the UK healthcare system, particularly in the National Health Service (NHS), where workforce shortages necessitate recruitment from abroad. Furthermore, there is a need to emphasise the importance of recognising the contributions of these staff groups, which are often underappreciated in addressing a retention crisis within the UK health system (3, 4).

The UK Nursing and Midwifery Council reported a substantial decline in international recruitment in 2024–2025, with first-time registrations from internationally educated professionals falling by 30.2% compared with the previous year (1). This reduction was particularly evident among recruits from India, the Philippines, and Nigeria, alongside increasing international attrition and a greater focus on domestic workforce development (1). The RCN's “Left Behind” Report (2025) suggests that investment in nursing career progression, supported by workforce planning, training, and clear career pathways, can improve recruitment, retention, leadership, and staff engagement (5). These mechanisms, in turn, are likely to contribute to better patient outcomes, stronger team performance, and organisational benefits, including reduced inequalities, improved productivity, and enhanced workforce sustainability (6). Since the Brexit referendum, the UK nursing and midwifery workforce has become increasingly reliant on internationally educated professionals from outside the EU/EEA. While recruitment from EU/EEA countries declined markedly after 2016, non-EU/EEA recruitment increased substantially, peaking in 2023–2024 before showing a modest decline in 2024–2025. Overall, the data highlights a significant shift in the source countries contributing to the UK nursing and midwifery workforce (7).

There is evidence of differential experiences among internationally educated nurses and midwives, linked to gender, race, language, and discrimination, that shape mobility decisions, settlement, and career trajectories in a post-Brexit UK (8–10). Nevertheless, the NHS continues to rely on international recruitment, particularly from non-EU countries, notably the Philippines, India and the African Continent, as well as other regions, to address persistent vacancies; this shift is tied to policy instruments, visa channels (Health and Care Worker visa), and broader immigration regimes established after Brexit (11). The UK's recruitment of IENs and IEMs has been a central component of workforce strategies for decades, driven by recognised shortages and policy aims to sustain services. This recruitment pattern has been described as a “train-for-export” model and as part of a broader international mobility of nurses and midwives, with substantial shares of new registrants historically originating from overseas (11, 12).

However, recognition of qualifications, language/testing regimes, settlement schemes, and targeted visa routes has had substantial implications for the recruitment, retention, and experiences of IENs and IEMs in the UK (13, 14). More so, ongoing monitoring of EU and non-EU health worker flows through professional registers (NMC, GMC) and employer recruitment data is essential to understand evolving patterns and to anticipate shortages in nursing and midwifery, particularly in the context of post-Brexit immigration dynamics (8, 13). Despite the post-Brexit challenges faced by IENs and IEMs, their contribution to the UK health workforce remains substantial and increasingly visible (11).

It has been reported that many IENs face career stagnation and limited leadership opportunities (15), but there is little evidence detailing the experiences of IEMs in the UK. Existing research focuses on migration experiences and integration challenges but lacks a comprehensive synthesis of their longitudinal career trajectories, leadership representation, and professional growth prospects (16). While IEMs are acknowledged as a globally mobile professional workforce, the existing literature from countries outside the UK highlights a gap in the understanding of their adaptation and career development processes in the UK (17). Therefore, more research attention should be given to IEMs (18).

The landscape for career progression and leadership for IENs and IEMs in the UK is likely to be shaped by numerous systemic and complex barriers that may stifle career advancement (19). While the NHS Long Term Workforce Plan (20) acknowledges internationally educated professionals as a vital component of the nursing and midwifery workforce, it highlights the importance of creating fair and supportive environments that enable their progression. IENs and IEMs often face challenges from the outset in relation to recognition and integration within the UK healthcare system (21), which undermines their previous qualifications and experiences in their native country, but also raises concerns about the potential de-skilling of these professionals when early recognition for these skills effectively stalls their career advancement (16). Moreover, Vamadevan (22) suggests that systemic and structural barriers such as a lack of mentorship and supportive networks in the UK healthcare system further complicate career advancement for this staff group. Limited visibility of IEN and IEM leaders, scholarship opportunities, and the focus on the global majority staff are required to support the strategies for career advancement and leadership (23).

Limited funding for advanced training, insufficient support from supervisors and mentors, and difficulties in balancing clinical and research responsibilities, all are reported to contribute to career stagnation (24, 25). Therefore healthcare systems that fail to implement supportive policies and professional development opportunities can create additional obstacles for IENs and IEMs aspiring to leadership positions (24). In response, Iheduru-Anderson (26) advocates expanding mentorship programmes, accessible leadership training, and equitable promotion policies to support career advancement and leadership integration within this staff group.

The UK midwifery career structure includes various advanced roles (consultant midwife, specialist midwife, clinical academic midwife, etc.), and recent work documents a recognised need for formal pathways to progression, including educational and organisational support, to translate clinical experience into expanded scopes of practice, including rapid progression to Band 6 pay grade as a specialist area of practice (27). IEMs similarly encounter unique challenges that impact their professional journey in the UK. The integration of IEMs into the UK workforce has been characterised by disparities in recognition of qualifications obtained abroad, which can limit their career prospects (28). IEMs often encounter barriers in certification processes that may not adequately recognise their international qualifications, leading to delays and obstructions to professional practice (29). Evidence indicates that international midwifery entrants often need to reframe their practice to align with UK norms of autonomy and decision-making, while navigating differences in the scope of practice and hierarchical relationships with obstetricians. This identity negotiation affects confidence, perceived autonomy, and long-term attachment to UK maternity services, and is influenced by the level of regulatory clarity and local workplace culture (30, 31). Although the UK has established advanced-practice roles, access for IEMs to formal progression pathways often depends on organisational culture, leadership support, and opportunities for engagement in higher education. The literature highlights the need for explicit career frameworks in midwifery, with pathways that link clinical expertise to leadership, research, and education roles (27, 31, 32).

This review explored the barriers to effective career progression and leadership opportunities for internationally educated nurses and midwives (IENMs) in the UK, stemming from systemic, structural, and cultural obstacles. Understanding these challenges is essential for developing targeted interventions that facilitate their integration, career advancement, and leadership, and address the unique needs of IENMs in the UK.

### Review question

The systematic review was undertaken to answer the following research question: What are the barriers and facilitators to career progression for internationally educated nurses and midwives in the UK?

## Methodology

This mixed methods systematic review was conducted in accordance with the Joanna Briggs Institute (JBI) methodology for mixed methods systematic reviews (MMSRs) (33), enabling the integration of diverse forms of evidence to capture both the breadth and depth of career-related experiences among IENMs in the UK. The protocol was registered with PROSPERO (CRD420251006645).

### Inclusion criteria

#### Population

This review considered studies involving IENs and IEMs who who obtained their primary nursing or midwifery qualification outside the UK and are working or registered with the NMC to practise in the UK.

Studies focusing exclusively on UK-trained nurses or midwives, other healthcare professionals, and undergraduate nursing or midwifery students, were excluded.

#### Phenomena of interest

This review considered studies that investigated barriers and/or facilitators to career progression, including promotion, leadership opportunities, professional development, mentorship, career advancement, pay progression, access to education or training, career mobility, or progression into specialist or senior roles.

Studies focusing solely on initial recruitment, migration processes/experiences, language testing, adaptation programmes, or registration processes without reference to career progression were excluded.

#### Context

This review considered studies conducted within healthcare settings in the UK, including NHS organisations, private healthcare settings, community care, social care, higher education, and independent healthcare sectors across England, Scotland, Wales, and Northern Ireland.

Studies were excluded if they were conducted outside the UK or where there was no clearly identifiable UK healthcare or professional context.

#### Types of studies

This review considered quantitative, qualitative and mixed methods studies. Quantitative studies included both experimental and quasi-experimental study designs, including randomised controlled trials, non-randomised controlled trials, before and after studies and interrupted time-series studies. In addition, analytical observational studies including prospective and retrospective cohort studies, case-control studies and analytical cross-sectional studies were considered for inclusion. This review also considered descriptive observational study designs including case series, individual case reports and descriptive cross-sectional studies for inclusion.

Qualitative studies included, but not limited to, designs such as phenomenology, grounded theory, ethnography and action research. The review also considered qualitative data reported within quantitative surveys for inclusion, where open questions relating to the phenomena of interest were asked.

Mixed method studies were only considered if data from the quantitative or qualitative components could be clearly extracted.

#### Search strategy

The search strategy aimed to identify both published and unpublished studies. A comprehensive search was conducted across five electronic databases including CINAHL Complete (EBSCO), MEDLINE (EBSCO), PubMed, Cochrane Library, and Ovid (Embase). The text words contained in the titles and abstracts of relevant articles, and the index terms used to describe the articles were used to develop a full search strategy (Supplementary Appendix 1). The search strategy, including all identified keywords and index terms were adapted for each included information source. In addition to academic databases, governmental reports and high-quality healthcare publications (such as those produced by NHS England, the Department of Health and Social Care, and the Royal College of Nursing) were searched manually via official websites and organisational repositories to capture relevant grey literature and policy documents. To ensure comprehensive coverage, Google Scholar was also searched to identify additional studies not indexed in major databases, and the reference lists of all included articles and relevant reviews were examined to identify further eligible sources.

The search was restricted to publications published between January 2005 and March 2025 to ensure that findings reflected contemporary workforce and policy developments within the UK healthcare system. This timeframe captures two decades of significant NHS workforce transformation, including the implementation of the Equality Act (2010), the NHS Long Term Plan (2019), and the NHS People Plan (2020), all of which have directly influenced recruitment, retention, and leadership development strategies for IENs and IEMs in the UK. Only studies published in English were considered due to language limitations and the researchers' inability to translate non-English sources.

#### Study selection

The initial database searches and citation tracking were performed by the first author (RS). Titles and abstracts were then screened by all three reviewers (RS, SB, CM) to assess compliance with the review's inclusion criteria, and duplicates were removed. Potentially relevant studies were retrieved in full and assessed in detail against the inclusion criteria. Full-text studies that did not meet the inclusion criteria were excluded, and any disagreements between the reviewers were resolved through discussion.

#### Assessment of methodological quality

Eligible studies were independently critically appraised by all three reviewers (RS, SB, CM) for methodological quality using the JBI Critical Appraisal Checklist for Qualitative Research for studies that featured qualitative methodologies and the Mixed Methods Appraisal Tool (MMAT) for those using a mixed-methods design (34). Any discrepancies between reviewers were resolved through detailed discussion until consensus was reached.

No studies were excluded based solely on methodological quality. This approach is consistent with JBI guidance for mixed methods systematic reviews, which recognises that studies of varying methodological quality may still contribute valuable contextual, experiential, and practice-based insights relevant to the review question. Excluding studies on this basis alone, due to methodological limitations, may result in the loss of important evidence relating to barriers and facilitators influencing career progression among IENMs in the UK.

#### Data extraction

Quantitative and qualitative data were extracted from included studies by two independent reviewers (RS, SB) using the standardised JBI data extraction tools in JBI SUMARI. The data extracted included specific details about the population, study methods, the phenomenon of interest, context, and outcomes relevant to the review question(s). Specifically, quantitative data comprised data-based outcomes of descriptive and/or inferential statistical tests. In addition, qualitative data comprised of themes or subthemes with corresponding illustrations, which were assigned a level of credibility by the first reviewer (RS) using the three levels as described by the standardised JBI qualitative data extraction tool:

1. Unequivocal (U) - evidence beyond a reasonable doubt, which may include conclusions that are matters of fact, directly reported/observed and not open to challenge.
2. Credible (C) - those conclusions that are, albeit interpretations, plausible in light of the data and theoretical framework.
3. Not Supported - when findings lack sufficient supporting evidence.

These were then discussed amongst the three reviewers (RS, SB, CM), resulting in a general consensus on the allocation of these levels (Supplementary Appendix 2). “Not Supported” data was excluded from the synthesis of data.

#### Data transformation

The quantitative data was converted into “qualitised data”. This involved transformation into textual descriptions or narrative interpretation of the quantitative results from the mixed methods studies, in a way that answered the review questions by repeated detailed examination (Supplementary Appendix 3).

#### Data synthesis and integration

The convergent integrated approach according to the JBI methodology for mixed methods systematic review using JBI SUMARI was used in this review (35). This involved assembling the “qualitised” data with the qualitative data. The assembled data were categorised and pooled based on similarity in meaning to produce a set of integrated findings in the form of line-of-action statements.

To enhance transparency and demonstrate the analytical process underpinning the synthesis, the development of the integrated themes and sub-themes is presented in Supplementary Appendix 2. Using the JBI convergent integrated approach enabled the quantitative evidence to indicate the prevalence, frequency, or scale of career-related inequalities, while the qualitative evidence explained how and why these inequalities were produced and experienced within healthcare organisations (33, 36).

## Results

### Study inclusion

The searches initially identified 1,357 records through database searching and two through citation searching and grey literature, resulting in 1,359 potentially relevant records. On further examination of the study titles, 316 of these records were excluded for duplication. The titles and abstracts were reviewed for the remaining records, and a further 1,032 were excluded for various reasons, including, opinion papers, those not focusing on the phenomena of interest or not meeting the inclusion criteria. The remaining 11 articles were retrieved for a full review, following which, three were excluded based on the agreed inclusion and exclusion criteria (systematic review = 2, literature review = 1), leaving eight articles to be examined for methodological quality (see Figure 1).

**Figure 1:**
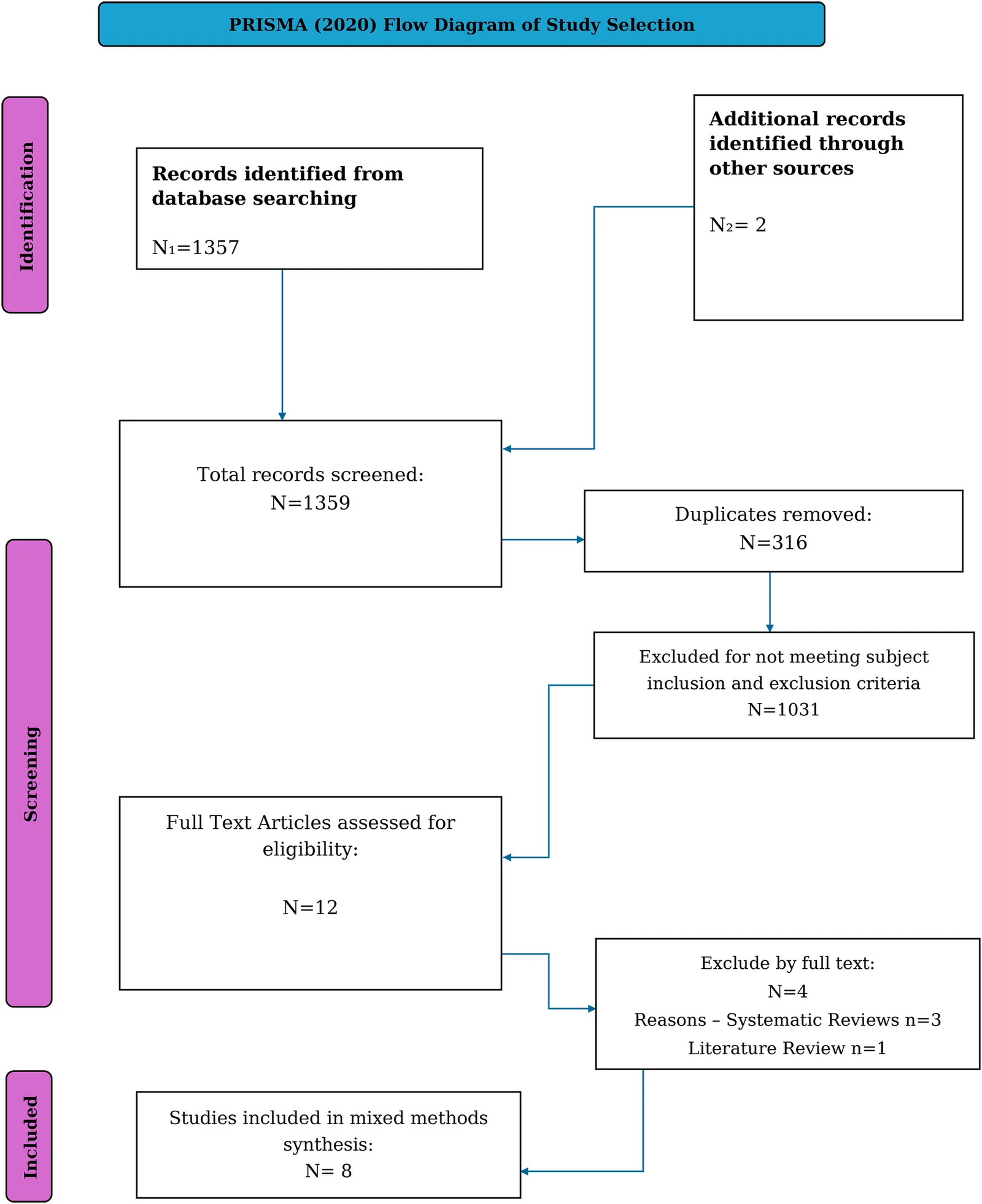
PRISMA flow diagram demonstrating N for each stage of the screening process (37).

### Methodological quality

Methodological quality of the eight included papers was determined by the relevant appraisal instrument. The JBI Critical Appraisal Checklist for Qualitative Research provided a framework for scoring the quality of qualitative studies, and the Mixed Methods Appraisal Tool (MMAT) for those using a mixed-methods design (see Tables 1, 2).

**Table 1:** Critical appraisal results for included studies using the JBI critical appraisal checklist for qualitative studies and qualitative components of mixed methods studies.

Author (Year)	Q1	Q2	Q3	Q4	Q5	Q6	Q7	Q8	Q9	Q10	Total
Adhikari and Melia (2015) (19)	Y	Y	Y	Y	Y	Y	N	Y	Y	Y	9/10
Blake (2025) (43)	U	Y	U	U	Y	U	N	U	N	Y	3/10
Henry (2007) (38)	Y	Y	Y	Y	Y	Y	U	Y	N	Y	8/10
Leone et al. (2020) (39)	Y	Y	Y	Y	Y	U	N	Y	U	Y	7/10
Terry and Spendlove (2025) (42)	Y	Y	Y	Y	Y	Y	Y	Y	Y	Y	10/10

Yes, Y; No, N; NA, Not applicable; U, Unclear.

1. Is there congruity between the stated philosophical perspective and the research methodology?.

2. Is there congruity between the research methodology and the research question or objectives?.

3. Is there congruity between the research methodology and the methods used to collect data?.

4. Is there congruity between the research methodology and the representation and analysis of data?.

5. Is there congruity between the research methodology and the interpretation of results?.

6. Is there a statement locating the researcher culturally or theoretically?.

7. Is the influence of the researcher on the research, and vice- versa, addressed?.

8. Are participants, and their voices, adequately represented?.

9. Is the research ethical according to current criteria or, for recent studies, and is there evidence of ethical approval by an appropriate body?.

10. Do the conclusions drawn in the research report flow from the analysis, or interpretation, of the data?.

**Table 2:** Critical appraisal results for included studies using the MMAT for mixed methods studies (34).

Study	S1	S2	5.1	5.2	5.3	5.4	5.5
Adhikari et al. (40)	Yes	Yes	Yes	Yes	Yes	Can't Tell	Yes
Pressley et al. (15)	Yes	Yes	Yes	Yes	Yes	Yes	Yes
Pressley et al. (41)	Yes	Yes	Yes	Yes	Yes	Can't Tell	Yes

Screening Questions (for all study types).

S1 Are there clear research questions?.

S2 Do the collected data allow the research questions to be addressed?.

MMAT Criteria for Mixed Methods Studies.

5.1. Is there an adequate rationale for using a mixed methods design to address the research question?.

5.2. Are the different components of the study effectively integrated to answer the research question?.

5.3. Are the outputs of the integration of qualitative and quantitative components adequately interpreted?.

5.4. Are divergences and inconsistencies between quantitative and qualitative results adequately addressed?.

5.5. Do the different components of the study adhere to the quality criteria of each tradition of the methods involved?.

### Characteristics of included studies

Further details on the characteristics of the included studies are presented in Table 3. Study designs were predominantly qualitative (*n* = 5), while three studies employed mixed methods designs.

**Table 3:** Characteristics of included studies.

Study	Country & Setting	Methodology	Participants	Geographic Context	Phenomena of Interest
Adhikari and Melia (19)	UK (Various sectors of healthcare)	Qualitative - In-depth interviews between 2006 and 2009 using snowball sampling	Nepali Migrant Nurses (*N* = 21)	Various sectors across the NHS.	Exploring wider workforce plans and career pathways
Adhikari et al. (40)	UK, NHS Trusts	Mixed-methods -Survey and Interviews	IENs (*N* = 21 for interviews), Band 5–8+	NHS hospitals across the UK	Career progression experiences, perceived inequities, support systems, and discrimination
Blake (43)	UK Higher Education and healthcare settings	Qualitative -Reflective Narrative Methodology	*N* = 605 Mentees	Academic and various healthcare settings	Diversity of mentoring Experiences from healthcare professionals from the UK, Europe, the Middle East, Africa, Asia, and Australasia.
Henry (38).	UK	Qualitative - Semi-structured interviews	*N* = 21 IENS from Ghana	Across NHS hospitals	Cultural adjustment, de-skilling, discrimination, and communication barriers
Leone et al. (39)	UK (NHS & health education organisations)	Qualitative -Semi-structured interviews	IENs from EU countries	NHS Trusts and recruiting organisations	Nurse mobility, integration, social/professional support systems
Pressley et al. (15)	England, NHS	Mixed-methods - Quantitative data and Thematic Interviews	IENs (*N* = 21), 6–26 years NHS experience	NHS Trusts	Career trajectories, progression from Band 5–8, enablers and barriers
Pressley et al. (41)	England, NHS	Mixed-methods survey	IENs (*N* = 773) < 2 years in UK	7 NHS England regions	Early migration experience, retention, personal/professional integration
Terry and Spendlove (42)	England, NHS maternity services	Qualitative -Interpretative Phenomenological Analysis	Band 6 midwives (*N* = 7)	NHS Trusts	Access to development, job satisfaction, and the influence of institutional support

The eight included studies were published between 2007 and 2025 (15, 19, 38–43), reflecting growing interest in the experiences, integration, and career development of IENMs within the UK workforce. Of the eight studies, six focused exclusively on nurses (15, 19, 38, 39, 41, 43), one focused exclusively on midwives (42), and one included both nurses and midwives (40).

Across the studies, a minimum of 1,752 participants were represented, comprising approximately 1,745 nurses and 7 midwives, although some studies did not fully disaggregate professional groups or participant characteristics. Participants originated from a diverse range of countries and regions, including Nepal (19), Ghana (38), European Union countries (39), and countries across the Middle East, Africa, Asia, and Australasia (43), illustrating the global nature of the UK nursing and midwifery workforce. The studies were undertaken across a variety of healthcare settings, predominantly within NHS hospitals and NHS Trusts (15, 38, 40–42), but also included healthcare education organisations and higher education institutions (39, 43), as well as broader healthcare sectors across the UK (19).

Collectively, the studies examined a range of phenomena, including career progression, workplace discrimination, integration, retention, mentoring, professional development, leadership opportunities, and organisational support for internationally educated nurses and midwives (15, 19, 39–43).

### Findings of the review

Taken together, the themes and subthemes (Supplementary Appendix 2) show that the majority of findings relate to IENs with limited evidence for IEMs. Nevertheless, the mixed-methods evidence was broadly convergent. Quantitative findings demonstrated the scale or frequency of several problems, including slower promotion, concentration at Band 5, absent career discussions and limited social networks, while qualitative findings explained how these patterns were experienced and reproduced within organisations. Furthermore, the findings indicate that IENMs' career progression is shaped by several interrelated factors, including recognition of prior experience, access to development, managerial support, promotion processes and workplace culture (15, 19, 38, 41). Under-recognition of previous expertise, skill mismatch, limited career planning, unequal access to CPD and experiences of bias may cumulatively delay advancement (15, 19, 38, 42). Conversely, structured induction, mentorship, sponsorship, leadership development and inclusive organisational support can facilitate professional integration and progression (39–41, 43) (See Table 4).

**Table 4:** Summary of integrated themes and sub-themes.

Theme	Sub-theme	Description of the issue	Supporting studies	Barrier, facilitator or both
Career Progression and Structural Barriers	Delayed and slower career progression	IENMs experienced slower promotion and were less likely to progress beyond lower NHS pay bands despite previous experience and professional capability.	(15, 19, 38)	Barrier
	Opaque and discretionary promotion processes	Promotion was perceived as lacking transparency and being influenced by managerial discretion, informal preparation, patronage and subjective assessments of leadership potential.	(15, 38)	Barrier
	Unequal access to career-development opportunities	Access to training, career planning, CPD and development roles varied between staff and was frequently dependent on local practices or managerial relationships.	(15, 41, 42)	Both
	Targeted leadership-development interventions	Leadership programmes increased confidence, visibility and readiness to pursue promotion, although they did not independently remove wider structural barriers.	(40)	Facilitator
Early-Career Scaffolding and Development Support	Structured induction and transition support	Comprehensive induction, effective recruitment and practical organisational support helped IENMs understand NHS expectations and adapt to unfamiliar professional environments.	(39, 41)	Both
	Career planning and developmental conversations	Early and continuing career discussions helped identify existing expertise and clarify progression pathways; their absence created uncertainty and delayed development.	(41, 42)	Both
	Continuing professional development and professional identity	Equitable CPD strengthened confidence, professional identity, career commitment and retention, while limited access contributed to dissatisfaction and skill underuse.	(19, 42)	Both
	Supportive line management and organisational support	Managers influenced access to induction, feedback, learning and progression. Consistent support facilitated development, whereas selective or inadequate support restricted opportunity.	(39, 40, 41, 42)	Both
Skill Mismatch and De-skilling	Under-recognition of previous expertise	Overseas qualifications, clinical experience and leadership capabilities were not consistently recognised within UK employment and career structures.	(38, 41)	Barrier
	Skill mismatch and professional de-skilling	Some IENMs were allocated roles and duties below their level of expertise, resulting in underused skills, reduced confidence and erosion of professional identity.	(19, 38)	Barrier
	Recognition and utilisation of prior competence	Recognition of transferable skills and previous professional achievements supported successful integration, appropriate role placement and subsequent development.	(39, 41)	Facilitator
Mentoring, Sponsorship and Leadership Access	Mentorship and professional guidance	Mentoring provided career guidance, reflective support, organisational knowledge and confidence, particularly during transition or career advancement.	(19, 40, 43)	Facilitator
	Sponsorship and managerial advocacy	Senior advocacy increased visibility and access to leadership opportunities; however, selectively distributed sponsorship reinforced patronage and inequity.	(38, 40, 42)	Both
	Leadership development and role modelling	Leadership programmes supported progression, while IENMs who reached senior roles became visible role models and potential sources of support for others.	(15, 19, 40)	Facilitator
Organisational Culture and Inclusion	Inclusive culture, psychological safety and belonging	Inclusive leadership and a sense of being valued strengthened confidence, teamwork, engagement, wellbeing and organisational commitment.	(40, 41)	Facilitator
	Discrimination, bias and exclusion	Bias, favouritism, culturally subjective expectations and unequal access to opportunities undermined confidence and discouraged further applications for promotion.	(15, 19, 38)	Barrier
	Organisational, social and practical support	Professional networks, peer relationships, housing support, family circumstances and financial security influenced integration, wellbeing and intentions to remain.	(39, 41, 42)	Both

Differences across studies related mainly to context and emphasis rather than direct contradiction. Earlier studies gave greater prominence to patronage, discrimination, de-skilling and opaque promotion practices (19, 38). In contrast, more recent studies also identified examples of inclusive leadership, structured development programmes and improved transition support (39–41). Positive outcomes were reported where targeted organisational interventions were available, particularly leadership development, induction and mentoring; however, access to these forms of support remained inconsistent and was often influenced by local management practices (40–42). Mentorship was generally beneficial in strengthening confidence and professional development (40, 43), but appeared insufficient where sponsorship, transparent promotion criteria and equitable development opportunities were absent (38, 42). Similarly, CPD and line-management support operated as facilitators when consistently available, but became barriers when access was limited or selectively allocated (41, 42).

Using the JBI convergent integrated approach, the qualitative and qualitised findings from the eight included studies were grouped by similarity of meaning into five integrated themes and associated sub-themes. Table 4 summarises the issue represented by each sub-theme, the studies contributing evidence and whether the issue operated as a barrier, facilitator or both.

For this review, a barrier was defined as any structural, institutional, social or individual factor that obstructed, delayed or restricted IENMs' access to career progression, professional development or leadership opportunities. Examples included opaque promotion systems, discrimination, under-recognition of expertise, de-skilling and unequal access to CPD (15, 19, 38, 42). A facilitator was defined as any factor that enabled, supported or accelerated professional integration, career development or progression into leadership, including structured induction, recognition of prior experience, mentorship, sponsorship, CPD and inclusive leadership (39–41, 43). Factors classified as “both” operated as facilitators when consistently and equitably available but became barriers when absent, limited or selectively provided.

1. Career Progression and Structural Barriers

Quantitative evidence demonstrated that IENs progressed more slowly than domestically educated nurses, particularly during the transition from Band 5 to Band 6. Pressley et al. (15) reported a median time to promotion of 6.8 years for internationally educated nurses compared with 5.8 years for UK-trained colleagues, with fewer IENs progressing beyond Band 6. These findings were supported by qualitative evidence describing progression pathways as unclear, inconsistent, and lacking transparency (16).

Promotion processes were also perceived as opaque and influenced by informal organisational practices. Henry (38) described promotion as shaped by managerial discretion, interview preparation, patronage and subjective assessments of leadership and communication, while Pressley et al. (15) identified bias and unequal access to opportunities as further barriers. These findings indicate that inequity may arise not only from formal criteria but also from unequal access to endorsement, organisational knowledge and developmental support.

Henry (38) identified organisational practices that hindered promotion, with participants describing opaque recruitment processes, inconsistent feedback, and limited opportunities to compete fairly for senior roles. Rather than attributing delayed progression to individual capability, participants perceived promotion as influenced by organisational discretion and informal decision-making. One participant described the process as:

“They tell us to sell ourselves, but then what are we saying to sell ourselves? What should we say to sell ourselves? (It) is our downfall because we don't know what to say and this is a time that we need help. How do you sell yourself … We need somebody to tell us that this is how the panel want it and then we will say it because we've got it there. But it's not forthcoming” (38)

These perceptions contributed to frustration, reduced motivation, and career stagnation, with several participants reporting that repeated unsuccessful promotion attempts discouraged them from pursuing further advancement.

The findings also demonstrated that progression depended on equitable access to organisational opportunities. Pressley et al. (15) identified unequal access to development opportunities and organisational recognition as important determinants of advancement, while Terry and Spendlove (42) found that limited opportunities for professional development reduced job satisfaction. Similarly, Pressley et al. (41) highlighted the importance of clear career pathways during the first two years of employment, yet many internationally educated professionals reported inconsistent career planning and limited access to structured progression support. Adhikari and Melia (19) further showed that limited development opportunities could prolong skill underuse and restrict movement into more appropriate roles.

Although several studies identified organisational initiatives designed to improve advancement, these were presented as mechanisms for addressing rather than eliminating structural barriers. Adhikari et al. (40) found that leadership development programmes increased confidence and created opportunities for career progression, suggesting that targeted organisational interventions can partially mitigate existing inequalities. Nevertheless, the integrated evidence consistently indicates that career progression is shaped not only by individual competence but also by organisational systems governing promotion, recognition, and access to opportunity.

2. Early-Career Scaffolding & Development Support

The evidence consistently identified comprehensive induction as a fundamental component of successful integration. Leone et al. (39) found that effective recruitment processes, employer support, and structured induction facilitated adaptation among migrant healthcare professionals across Europe. Similarly, Pressley et al. (41) reported that supportive line managers and comprehensive induction programmes eased transition into NHS practice, while organisational, professional, and personal support promoted successful workplace integration. These findings suggest that early support extends beyond clinical orientation to include social integration and familiarisation with organisational systems.

Access to CPD emerged as another essential component of early-career support. Terry and Spendlove (42) found that limited access to development opportunities reduced job satisfaction, whereas equitable access to CPD strengthened professional identity, increased career commitment, and encouraged retention. Participants described opportunities for development as dependent on managerial support, with inconsistent access creating uncertainty about future career prospects. Similarly, Pressley et al. (41) found that fewer than half of respondents had participated in formal career-development conversations during their first two years of employment, indicating that structured career planning was not routinely embedded within early employment.

Qualitative findings further illustrated how inconsistent developmental support affected professional confidence. Terry and Spendlove (42) reported that annual appraisals and career discussions were frequently perceived as insufficient to guide career progression, while managerial discretion often influenced access to learning opportunities. Participants who received regular developmental feedback and supportive supervision described greater motivation, stronger professional identity, and increased intention to remain within the organisation. Conversely, inconsistent support contributed to uncertainty, disengagement, and reduced confidence in career advancement.

The importance of structured developmental support was reinforced by evidence highlighting the risks associated with its absence. Adhikari and Melia (19) demonstrated that IENs who entered roles with limited opportunities for professional development experienced prolonged periods of skill underutilisation and delayed career progression. Although these findings are explored further within the theme of Skill Mismatch and De-skilling, they also illustrate the importance of early support in enabling internationally educated professionals to translate existing expertise into meaningful career development within the NHS.

Overall, the integrated findings indicate that early-career scaffolding extends beyond induction alone. Structured orientation, equitable access to CPD, regular career-development conversations, supportive line management, and ongoing professional guidance collectively facilitate integration, strengthen professional identity, and support retention among IENs and IEMs. Where these mechanisms were inconsistent or absent, participants described slower adaptation, reduced confidence, and diminished expectations for career progression.

3. Skill Mismatch and De-skilling

The qualitative evidence consistently demonstrated that many internationally educated professionals entered the UK workforce with substantial clinical knowledge and experience but were employed in roles that failed to utilise these capabilities. Adhikari and Melia (19) provided a detailed ethnographic account of Nepali nurses recruited to the UK expecting opportunities to develop their nursing careers but instead finding themselves undertaking routine personal care within private residential care homes. Participants described this mismatch between expectations and reality as professionally disempowering and detrimental to their confidence.

One participant explained:

“..but when I arrived here, it was all about only pad round [routine change of incontinent pads for dependent elderly people], basic wash, feeding people and putting them in bed. I found it completely different from what I had thought.” (19)

Another participant reflected on the loss of professional identity associated with this experience:

“I was supposed to be doing my adaptation training, but I was learning to set out the dinner trolley, offer and collect menus, serve food and feed older adults.. it felt as if I was doing hotel management training, not learning any clinical nursing at all.” (19)

These accounts illustrate how skill mismatch extended beyond occupational dissatisfaction, resulting in the erosion of clinical competence, diminished professional identity, and reduced confidence. For some participants, prolonged employment in roles that underutilised their expertise led to feelings of entrapment and uncertainty about future career aspirations.

Evidence from other studies reinforced these findings. Henry (38) reported that overseas qualifications and previous professional experience were frequently undervalued within NHS career structures, limiting opportunities to progress into more senior roles. Similarly, Pressley et al. (41) found that many IENs remained employed at Band 5 despite substantial previous experience, suggesting that prior qualifications and expertise were not consistently recognised within NHS career pathways.

While the qualitative findings highlighted the lived experience of de-skilling, the quantitised evidence demonstrated its wider organisational consequences. Pressley et al. (15) identified slower progression among internationally educated nurses compared with UK-trained colleagues, while Pressley et al. (41) reported that prior skills and qualifications were frequently under-recognised during career development. In contrast, Leone et al. (39) found that professional achievements and recognition of existing expertise enhanced migration success, suggesting that effective utilisation of prior skills facilitates both professional integration and long-term retention.

Collectively, the evidence indicates that skill mismatch begins at the point of workforce entry through inadequate recognition of previous qualifications, inappropriate role allocation, and limited opportunities to apply existing expertise. These experiences contribute to de-skilling, delayed career progression, and reduced professional confidence, whereas recognition of prior competence supports successful integration and career development.

4. Mentoring, Sponsorship, and Leadership Access

Across the included studies, mentoring and sponsorship emerged as distinct but complementary strategies that increased professional confidence, visibility, and access to career advancement. The evidence consistently demonstrated that mentoring supported professional development by providing guidance, encouragement, and opportunities for reflective learning. Blake (43) found that mentoring enhanced confidence, improved career readiness, and reduced barriers experienced by under-represented groups. Similarly, Adhikari et al. (40) identified mentors and supportive line managers as important facilitators of professional development, helping internationally educated professionals understand organisational expectations and navigate NHS career pathways.

However, the findings also distinguished mentoring from sponsorship. Whereas mentoring focused on personal and professional development, sponsorship involved senior colleagues actively advocating for individuals, creating opportunities for leadership development, and increasing visibility within organisational networks. Adhikari et al. (40) found that leadership development programmes incorporating sponsorship and inclusive leadership increased confidence and created opportunities for career progression. Participants described feeling more prepared to pursue leadership roles when senior staff actively endorsed their capabilities and encouraged participation in development programmes.

The importance of organisational advocacy was reinforced by Henry (38), who described how access to career opportunities was frequently influenced by informal support from senior managers. One participant explained:

“They might even do a practice interview with you. So they prepare somebody. If a manager is preparing you, you know you stand a very good chance … (but) they need to interview two or three people for it to be fair or seem to be fair.” (38)

Although this quotation illustrates the unequal distribution of organisational support, it also demonstrates the influence that senior advocacy can have on career progression. Participants who lacked similar support perceived fewer opportunities to compete for promotion and described managerial relationships as an important determinant of access to development opportunities:

“Not everybody gets that sort of assistance… Some might, but not everybody gets it.” (38)

Evidence from Terry and Spendlove (42) similarly demonstrated that relationships with line managers influenced access to professional development and career opportunities, reinforcing the role of leadership in enabling progression. In contrast, Pressley et al. (15) highlighted the positive outcomes of successful progression, with IENs who advanced into leadership positions becoming influential role models for colleagues and contributing to more diverse leadership within healthcare organisations.

Overall, the integrated findings indicate that mentoring, sponsorship, and leadership development operate as important enablers of career progression. While mentoring builds confidence and professional capability, sponsorship provides access to influential networks and opportunities that facilitate advancement into leadership roles. Together, these mechanisms support more equitable career progression by increasing visibility, strengthening professional confidence, and promoting leadership representation among IENs and IEMs.

5. Organisational Culture and Inclusion

Across the included studies, organisational culture emerged as a fundamental influence on the experiences of IENs and IEMs, shaping their confidence, engagement, wellbeing, and intentions to remain within the workforce. The evidence consistently demonstrated that inclusive workplace cultures fostered a greater sense of belonging and professional value. Adhikari et al. (40) found that initiatives promoting inclusive leadership and awareness of unconscious bias enhanced confidence, teamwork, and organisational engagement among minority ethnic nurses and midwives. Participants emphasised that meaningful inclusion depended on leaders demonstrating inclusive behaviours in everyday practice rather than simply promoting diversity as an organisational value. As one participant explained, there was a need for “*leaders who listen and act, not just talk about diversity”* (40). This reflects the importance of psychological safety, whereby staff felt able to contribute, raise concerns, and engage confidently because leadership behaviours reinforced inclusion in practice.

Similarly, Pressley et al. (41) identified organisational, professional, and social support as essential components of successful integration. Participants who reported positive workplace experiences, supportive peer networks, and inclusive organisational environments also reported greater wellbeing, stronger organisational commitment, and increased intentions to remain within the NHS. Leone et al. (39) reinforced these findings, demonstrating that supportive organisational environments, alongside practical assistance such as housing, family support, and professional integration, positively influenced both retention and migration success among healthcare professionals.

Conversely, exclusionary workplace cultures undermined confidence and reduced engagement. Henry (38) described how experiences of discrimination, favouritism, and exclusion from opportunities contributed to frustration and disengagement. One participant reflected:

“Several nurses also reported that they were so traumatised or demoralised by the experience that they felt unable to seek feedback from the people they believed discriminated against them.. many withdrew from career development, ceased applying for promotion, and their careers stagnated.” (38)

Rather than representing isolated interpersonal experiences, these findings suggest that organisational culture influences how internationally educated professionals perceive fairness, belonging, and their future career prospects. When staff experienced exclusion or unequal treatment, confidence diminished and aspirations for career progression declined.

The findings also demonstrated that feeling valued by employers extended beyond interpersonal relationships to organisational investment in staff development. Terry and Spendlove (42) found that participants who perceived genuine organisational commitment to their professional development reported greater job satisfaction and stronger intentions to remain in the profession. Similarly, qualitised findings from Pressley et al. (41) indicated that inclusive organisational cultures promoted engagement, wellbeing, and retention, reinforcing the importance of supportive workplace environments throughout internationally educated professionals' careers.

Overall, the integrated findings demonstrate that organisational culture underpins many of the factors influencing career progression and retention among IENs and IEMs. Inclusive leadership, psychological safety, equitable treatment, and organisational support foster confidence, engagement, and belonging, whereas exclusionary cultures contribute to disengagement, reduced aspirations, and workforce attrition.

## Discussion

This mixed-methods systematic review demonstrates that the career trajectories of IENMs in the UK are predominantly shaped by structural, organisational, and cultural factors rather than by individual capability. Across the included studies, career progression emerged as a complex process influenced by organisational transparency, access to professional development, recognition of prior expertise, workplace inclusion, and leadership support. These findings extend previous evidence highlighting the institutional barriers experienced by migrant healthcare professionals and provide a contemporary synthesis of factors influencing career progression and retention within the NHS (15, 16, 18).

The review identified persistent structural barriers within promotion systems that continue to constrain equitable advancement. Quantitative evidence indicating slower progression through NHS pay bands among internationally educated staff reinforces longstanding concerns regarding inequitable promotion pathways (15). Qualitative findings provided further explanatory depth, revealing promotion processes that were frequently perceived as opaque, dependent upon informal networks, and influenced by discretionary managerial decision-making rather than transparent merit-based criteria. These findings are consistent with earlier work by Henry (38), who identified organisational gatekeeping and racialised perceptions of leadership suitability as important barriers to progression. Similar concerns regarding favouritism and inequitable access to opportunities have also been reported by Bapuji et al. (44) and Jang et al. (45), suggesting that progression inequities remain embedded within organisational cultures rather than being isolated incidents. Consequently, transparent promotion pathways, standardised progression criteria, and leadership accountability mechanisms are required to ensure that advancement is based upon capability and performance rather than informal sponsorship networks or managerial discretion.

The findings also highlight the continued challenge of skill mismatch and de-skilling among internationally educated practitioners. Consistent with the work of Adhikari and Melia (19), IENs and IEMs were frequently employed in roles that failed to utilise their previous qualifications, specialist expertise, or leadership experience. Such underutilisation can undermine professional confidence, delay progression into NHS roles, and shape long-term career aspirations. Leone et al. (39) similarly highlighted the consequences of inadequate recognition of overseas qualifications and experience. The findings therefore suggest a need for reform of credential-recognition systems and workforce deployment practices to ensure that internationally educated practitioners are placed in roles commensurate with their expertise. Such reforms would not only reduce de-skilling but also maximise workforce productivity and contribute to workforce retention.

A particularly important finding concerns the role of early-career scaffolding in supporting progression. Structured induction programmes, preceptorship, mentorship, and continuing professional development opportunities consistently emerged as important facilitators of integration and career development. The National Preceptorship Framework provides an important mechanism through which newly recruited international staff can be supported during their transition into UK practice (46). However, the evidence suggests considerable variation in implementation across organisations. Terry and Spendlove (42) identified inconsistent access to developmental feedback and CPD opportunities, while several studies reported progression becoming dependent on managerial support rather than systematic workforce development structures. These findings indicate that equitable access to protected CPD time, transparent appraisal processes, and structured career development planning should be embedded within organisational policy and monitored through workforce performance frameworks.

Mentorship emerged as a significant enabler of professional growth; however, the review demonstrates that mentorship alone is insufficient to address progression inequities. The strongest evidence for advancement was associated with sponsorship, inclusive leadership, and psychologically safe workplace environments (40, 43, 47). Sponsorship differs from mentorship in that it provides active advocacy, visibility, and access to developmental opportunities within organisational structures. The findings therefore support the implementation of formal, funded mentorship and sponsorship programmes specifically designed for internationally educated nurses and midwives. Such programmes have the potential to disrupt informal gatekeeping processes and enhance leadership readiness among internationally educated staff. Furthermore, structured sponsorship initiatives may contribute to greater representation of internationally educated professionals in senior clinical, managerial, educational, and research leadership positions.

The findings further demonstrate that organisational culture plays a pivotal role in shaping progression opportunities. Workplaces characterised by psychological safety, inclusivity, transparency, and supportive leadership were associated with greater professional confidence and willingness to pursue promotion opportunities (40, 43). Conversely, environments characterised by favouritism, exclusion, and unspoken organisational norms were associated with disengagement and withdrawal from progression pathways (44). These findings suggest that structural reforms alone are unlikely to achieve sustainable change without concurrent cultural transformation. Consequently, leadership development programmes should incorporate anti-racist leadership, cultural competence, unconscious bias awareness, and cross-cultural communication as core leadership competencies. Such an approach aligns with recommendations emerging from both the NHS Management and Leadership Framework (48) and broader workforce equity agendas.

The implications of these findings extend beyond individual career outcomes to broader questions of workforce sustainability, leadership diversity, and organisational legitimacy. Within the context of the Workforce Race Equality Standard (WRES) (49), the continued underrepresentation of internationally educated professionals in leadership positions raises concerns regarding organisational fairness and effectiveness. Fragkos et al. (50) argue that the underutilisation of diverse talent pools limits organisational innovation and leadership capacity. Similarly, Hammond et al. (51) and Chastney et al. (52) highlight the importance of representative leadership in maintaining trust among diverse communities and promoting equitable healthcare delivery. Therefore, progression inequities should not be viewed solely as workforce issues but as matters of healthcare quality, organisational credibility, and system performance.

The NHS Management and Leadership Framework (48) offers a timely policy mechanism through which many of the barriers identified within this review can be addressed. Its emphasis on inclusivity, integrity, accountability, and compassionate leadership resonates strongly with the themes identified across the evidence base. However, the effectiveness of the framework will depend upon consistent implementation and meaningful accountability mechanisms (53). Leadership competencies related to inclusion, talent development, and workforce equity should therefore be embedded within appraisal systems, promotion processes, and leadership performance reviews. Linking these measures to WRES outcomes would provide measurable indicators of progress while ensuring that workforce equity becomes a tangible leadership responsibility rather than an aspirational objective (54, 55).

The significance of these findings is amplified by contemporary workforce challenges. The Nursing and Midwifery Council (56) reported a substantial reduction in international recruitment, estimated at approximately 50%, highlighting the increasing importance of retaining and developing the internationally educated workforce already employed within the UK. At the same time, IENs and IEMs continue to face inequitable task allocation, limited progression opportunities, and inadequate recognition of prior expertise (29, 57). Evidence from Likupe and Archibong (58) further demonstrates how race intersects with migration status, with Black African nurses reporting higher levels of racial harassment than their peers. Similarly, Vamadevan et al. (23) identified significant barriers to research engagement and leadership participation among nurses from underrepresented backgrounds. Together, these findings illustrate how structural inequities continue to shape professional experiences across multiple domains of practice and leadership.

Beyond mentorship and sponsorship, the literature highlights leadership development and structured engagement in research as important mechanisms for enhancing professional visibility, influence, and career progression. Participation in research has increasingly been recognised as a pathway through which nurses and midwives develop leadership competencies, expand professional networks, and gain access to opportunities that support advancement into senior clinical, academic, and strategic roles. National initiatives such as the NICE Senior Nurse Research Leader Programme provide a useful example of how investment in leadership capacity and research engagement can strengthen the profile, influence, and impact of nursing and midwifery across healthcare systems (59). Although not specifically designed for IENs and IEMs, such initiatives offer a valuable model for addressing some of the systemic barriers identified within this review. Greater inclusion of IENMs within leadership and research development programmes may enhance professional visibility, strengthen evidence-informed practice, and create additional routes into leadership positions. Furthermore, increased participation in research can help challenge perceptions of limited capability, facilitate access to influential professional networks, and support more equitable representation within senior clinical, educational, managerial, and research leadership roles. In the context of ongoing concerns regarding progression inequities, healthcare organisations should consider targeted strategies to ensure that IENMs are actively encouraged and supported to engage in leadership and research development opportunities as part of broader workforce inclusion and talent management agendas (23, 59).

The findings of this review should also be interpreted within the broader international literature on health workforce migration, which suggests that the experiences of IENMs are shaped not only by local organisational practices but also by wider global labour market dynamics (21). International recruitment is often framed by destination countries as a pragmatic response to workforce shortages; however, the literature highlights that this model can reproduce dependency on migrant labour without necessarily ensuring equitable professional integration or career development once staff arrive in the host system (60). In this respect, the findings of the present review move beyond concerns about recruitment and transition, demonstrating that the long-term value of IENMs is constrained when systems fail to recognise prior expertise, provide transparent progression routes, or create inclusive leadership pipelines.

This interpretation is consistent with international evidence that IENs often experience a form of occupational downgrading or professional re-positioning following migration, particularly where regulatory, organisational, or cultural systems privilege domestically acquired experience over internationally gained competence (21, 41). While adaptation to a new regulatory and clinical context is necessary, the findings suggest that adaptation can become inequitable when it is conflated with a need to “start again” professionally (63). This distinction is important because it shifts the analysis from individual readiness or confidence to the structures through which competence is recognised, legitimised, and rewarded. The under-recognition of prior qualifications and leadership experience therefore represents not only a personal career barrier but also an inefficient use of international workforce capability (62).

The review also adds to international debates about the racialised and gendered nature of career progression in healthcare. Many IENMs migrate from countries in Africa, Asia, the Caribbean, and other regions of the global majority, meaning that migration status often intersects with race, ethnicity, language, accent, gender, and professional hierarchy. The barriers identified in this review, including opaque promotion processes, unequal access to development, reliance on informal sponsorship, and exclusion from leadership opportunities, therefore need to be understood as potentially intersectional rather than purely administrative. This is significant for policy because interventions focused only on generic career development may be insufficient unless they also address bias, discrimination, and the informal organisational norms through which opportunity is distributed.

From a policy perspective, the findings challenge workforce strategies that prioritise international recruitment without equal investment in retention, progression, and leadership development. In the context of reduced international recruitment and persistent NHS workforce shortages, failure to develop IENMs risks reinforcing a cycle in which overseas staff are recruited to fill vacancies but are not systematically supported to progress into senior clinical, educational, managerial, research, or strategic leadership roles. This has implications for workforce sustainability, morale, and organisational justice. It also limits the diversity of decision-making spaces and reduces the extent to which internationally educated staff can influence the design of services, workforce policy, and professional education. The NHS Long Term Workforce Plan (2023) and NHS People Plan (2020) both comit explicitly to inclusive, diverse, and sustainable nursing workforces. However, the findings of this review indicate a persistent gap between national policy commitments to inclusive workforce development and their translation into consistent organisational practice. Therefore, policy responses need to move beyond localised mentoring schemes or individual resilience-building approaches towards system-level accountability for equitable career progression, recognition of international expertise, and access to leadership development. Largely, the evidence suggests that achieving equitable career progression for IENMs requires a comprehensive and system-level response. Transparent progression pathways, equitable access to continuing professional development, recognition of international expertise, structured mentorship and sponsorship programmes, anti-racist leadership development, and robust monitoring of workforce equity outcomes represent interconnected strategies for addressing the barriers identified in this review. Such reforms align with the NHS Long Term Workforce Plan (20), the WRES (49), and the NHS Management and Leadership Framework (48). More importantly, they are essential for ensuring that the NHS fully benefits from the expertise, experience, and leadership potential of a workforce in which IENs and IEMs now comprise almost one-quarter of registrants (2). By moving beyond recruitment-focused strategies and investing in progression, inclusion, and leadership development, healthcare organisations can strengthen retention, improve workforce morale, and create a more equitable and sustainable NHS workforce for the future.

### Study limitations

This systematic review has several limitations that should be acknowledged when interpreting the findings. The primary limitation of the review was that the evidence base comprised only eight studies, which restricted the breadth of conclusions that could be drawn. This reflected the broad gap in evidence on the combination of IENs and IEMs. Moreover, the review highlighted the significant underrepresentation of IEM-specific research in the UK context, resulting in a reliance on studies that disproportionately centre nurses rather than midwives. Homer et al. (61) suggest a lack of targeted research that explicitly addresses the unique circumstances and challenges faced by IEMs, creating a significant knowledge gap that negatively affects practice and development in midwifery.

Another limitation relates to the heterogeneity of study designs. Several studies demonstrated limited reflexivity, minimal detail on researcher positionality, or incomplete reporting of analytic procedures (e.g., 38, 43), which may influence the credibility and transferability of findings. In addition, some older qualitative studies (e.g., 38) predate substantial changes in the NHS workforce and migration policy, meaning their relevance may be partially affected by temporal and policy shifts. Although the review's inclusion period (2005–2025) was selected to reflect two decades of NHS workforce transformation, this timeframe also spans major structural reforms (e.g., the Equality Act 2010, the NHS People Plan 2020, and the post-COVID recovery). These shifts create potential contextual variability that cannot be fully accounted for in comparing older and more contemporary studies. As a result, some earlier findings may no longer fully represent current institutional dynamics, while newer studies may not yet reflect long-term career trajectories.

Moreover, while mixed-methods synthesis allowed the integration of quantitative progression data with qualitative experiences, quantitative data on IEMs were particularly limited, as most large datasets (e.g., 15, 41) focused primarily on IENs. This constrained the ability to compare sectoral and professional differences (e.g., NHS vs. private, nursing vs. midwifery) in a systematic and statistically meaningful way. While these limitations do not undermine the validity of the review, they highlight key gaps in the current evidence base and underscore the need for further research, particularly longitudinal, midwifery-specific, and sector-comparative studies, to better understand the career trajectories and leadership opportunities for IENs and IEMs in the UK.

## Conclusion

This mixed-methods systematic review provides the first integrated synthesis of evidence examining the barriers and facilitators to career progression and leadership development among IENMs working in the UK. Across the eight included studies, the review highlights how structural, institutional, and cultural barriers, such as opaque promotion pathways, limited recognition of prior expertise, unequal access to CPD, and inconsistent mentorship and sponsorship, shape the professional trajectories of IENMs. The evidence demonstrates that career progression is hindered not by lack of capability, but by systemic arrangements that undervalue international experience, restrict development opportunities, and perpetuate inequitable organisational cultures. These findings reinforce earlier concerns raised in the introduction about the underutilisation of IENM expertise, the risk of de-skilling, and the long-standing gaps in research regarding internationally educated midwives. The convergent integrated synthesis further reveals striking consistencies across NHS and private sector settings, with IENMs in both environments encountering misalignment between skills and roles, constrained mobility, and limited access to supportive leadership structures.

In light of these findings, it is evident that meaningful progress requires system-wide reforms aligned with national policy commitments, such as the NHS Long-Term Workforce Plan and the emerging NHS Management and Leadership Framework. Implementing transparent promotion processes, strengthening mentorship and sponsorship pathways, and embedding inclusive leadership behaviours are critical steps toward creating equitable environments in which IENMs can flourish professionally. Furthermore, linking development opportunities and progression monitoring to frameworks such as the WRES will ensure that inclusion and fairness are not aspirational but measurable and accountable. As the UK continues to rely heavily on internationally educated professionals to sustain its health services, investing in their career advancement is not only a matter of equity but is essential for workforce stability, leadership diversity, and high-quality patient care. This review highlights the urgent need for further midwifery-specific and sector-comparative research to address remaining gaps and to inform policies that fully harness the talents of a diverse global workforce.

## Data Availability

The original contributions presented in the study are included in the article/Supplementary Material, further inquiries can be directed to the corresponding author.
