# Supplementary material for: Barriers and facilitators to career progression and leadership among internationally educated nurses and midwives in the UK: a mixed methods systematic review
Source: Front Health Serv. 2026 Oct 21;6:1854601. doi: 10.3389/frhs.2026.1854601 (PMC13736840; doi:10.3389/frhs.2026.1854601)
Supplement: Supplementary file 1 [file Supplementaryfile1.docx]

**Appendix – 1: Search strategy using the PICO framework**

| **PICO Element** | **Search Strategy** |
| --- | --- |
| **Population** | international nurse* OR internationally educated nurse* OR overseas nurse* OR foreign nurse* OR migrant nurse* OR international midwi* OR internationally educated midwi* OR migrant midwi* OR foreign-trained nurse* OR foreign-trained midwi* AND United Kingdom OR UK OR NHS OR England OR Scotland OR Wales OR Northern Ireland |
| **Intervention/Exposure** | leadership OR leadership development OR career development OR career progression OR promotion OR mentorship OR mentoring OR preceptorship OR continuing professional development OR CPD OR discrimination OR racism OR bias OR inclusion OR diversity OR workplace culture OR organisational culture OR professional development |
| **Comparator** | No comparator terms included. Comparisons between NHS and private sector, or between subgroups of internationally educated nurses and midwives, will be identified during screening rather than through search terms. |
| **Outcomes** | leadership role* OR promotion OR career advancement OR career progression OR career stagnation OR professional development OR access to training OR mentorship OR belonging OR retention OR job satisfaction OR experience* |
